# Manipulating brassinosteroid signaling pathway to genetically improve horticultural plants

**DOI:** 10.1007/s42994-025-00201-y

**Published:** 2025-02-22

**Authors:** Xiaopeng Li, Jiaxuan Li, Hossain M. Zabed, Junjie Li, Min Xiong, Hongyong Shi, Jia Li

**Affiliations:** Guangdong Provincial Key Laboratory of Plant Adaptation and Molecular Design, School of Life Sciences, Guangzhou University, Guangzhou, 510006 China

**Keywords:** Brassinosteroids, Fruit development, Horticultural plant, Leaf development, Plant hormone, Stress adaptation

## Abstract

Brassinosteroids (BRs), a class of plant-specific steroidal hormones, play crucial roles in regulating various plant physiological functions, such as growth, development, and adaptability to the environment. Despite this broader role of BRs, previously published reviews mainly focused on the molecular mechanisms of BR-mediated regulation of vegetative and reproductive growth of model plants like *Arabidopsis* and some food crops, such as rice, maize, and wheat. While horticultural plants hold significant economic importance in modern agriculture, less attention has been paid to understanding the role of BRs in regulating the physiological functions of these plants. Given the lack of relevant reviews, this article aims to discuss the major roles of BRs in horticultural plants, particularly fruit and leaf development, whole plant architecture, and adaptive stress response. We also highlight key challenges and provide some future research directions for genetically improving horticultural plants by altering the BR signaling pathway.

## Introduction

With the rapid growth of the world’s population, which is expected to reach 10 billion by 2050, ensuring sufficient food supplies and balanced diets is becoming a major concern, worldwide (FAO 2017). To address this issue, scientific communities across the world have devoted significant research efforts to achieve sustainable agriculture. Although these fundamental studies have significantly contributed to the improvement of crop yields and quality, the sustainability of agriculture is still threatened by global climate change. In addition, previous research mainly focused on cereal crops serving as the primary source of carbohydrates for human consumption, while relatively less attention has been paid to horticultural crops. These crops include a broad range of plants, such as vegetables, fruit trees, turfgrasses, ornamental plants, and aromatic and medicinal plants, which provide a diversified and nutrient-rich composition of the human diet. Among them, vegetables and fruits are particularly important because they are rich in nutrients and have a significant impact on global human health (Del Río-Celestino and Font 2020; Wallace et al. 2020). Research on horticultural crops can be classified into different subfields based on the specific nature and intended use, namely olericulture, fruticulture, floriculture, arboriculture, turfgrass science, and postharvest physiology. Given the growing population and the need for sustainable food production, it is imperative to pay greater attention to horticultural research to achieve food security and improved quality of life.

Plant growth and development are often regulated by a variety of phytohormones, with brassinosteroids (BRs) receiving significant research attention. BRs belong to a class of polyhydroxysteroids with more than seventy identified members (Bajguz and Tretyn 2003; Bajguz 2007; Zhao and Li 2012). BRs are widely distributed in the plant kingdom and have been found in dicots, monocots, gymnosperms, algae, and ferns (Vriet et al. 2015). Moreover, BRs occur in various parts of plants (Bajguz and Tretyn 2003). In higher plants, BRs can be biosynthesized in almost all tissues, but their concentrations vary between different organs or tissues, with the highest levels occurring in actively growing areas (Bajguz and Tretyn 2003; Vukašinović et al. 2021). Due to the inability of BRs to be transported over long distances (Symons and Reid 2004; Wang et al. 2023a), their activity relies primarily on their local concentration. This unique property has motivated scientists and breeders to selectively regulate BR content or signaling to achieve tissue-specific improvement in various crops.

BRs have been shown to play a crucial role in various aspects of plant growth and development, including skotomorphogenesis, photomorphogenesis, root growth and development, reproductive organ development, vascular tissue development, and environmental adaptation (Wang et al. 2012; Wei and Li 2016; Planas-Riverola et al. 2019; Nolan et al. 2020). In cereal crops, BRs are critical regulators of several important agronomic traits, such as plant height, lamina bending, tiller number, grain size, and yield (Zhang et al. 2014; Tong and Chu 2018; Lin 2020). For horticultural plants, BRs were also shown to regulate a variety of economically important traits, including the size, color, and ripening process of fruits, and the leaf morphology of vegetable crops (Zhang et al. 2022b; Yang et al. 2023b). To date, some review articles have provided detailed summaries of the role of BRs in the model plants *Arabidopsis* and rice (Tong and Chu 2018; Nolan et al. 2020). A systematic summary of the role of BRs in the regulation of horticultural traits is also urgently needed. The main goal of plant biology research is to uncover mechanistic details of plant growth and development, and to provide theoretical guidance for future molecular breeding. In this review, we summarize in detail the role of BR in regulating fruit and leaf development, overall plant architecture, and stress adaptation in horticultural plants, and discuss possible future applications of BRs in horticultural plant improvement.

## Biosynthesis and signaling pathway of BL

**Fig. 1:**
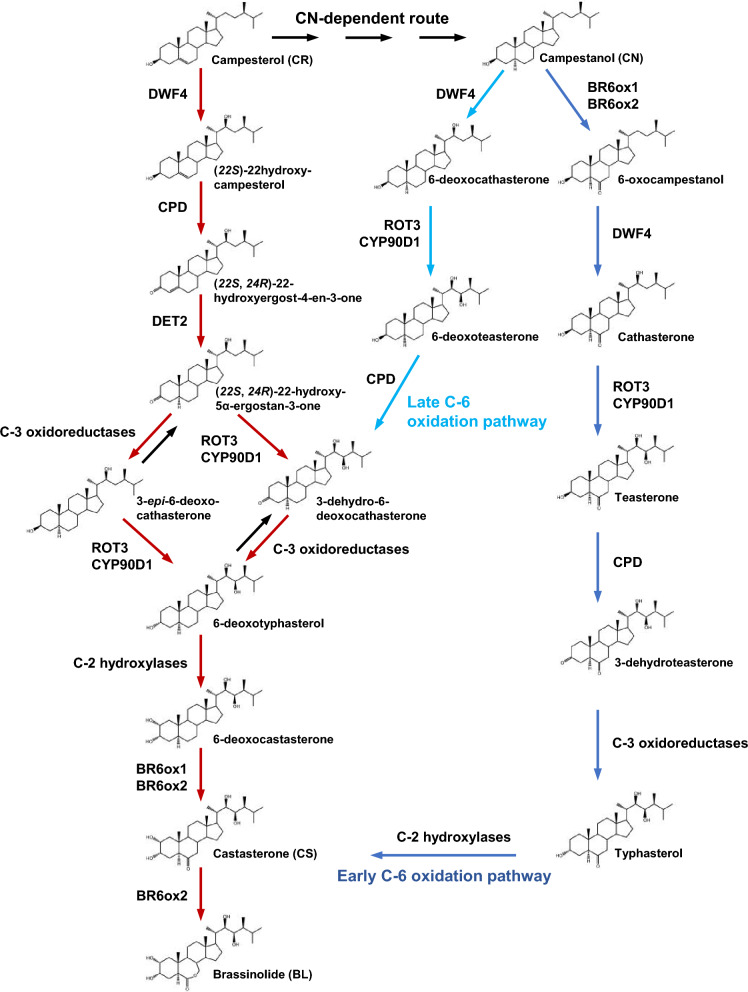
Simplified brassinosteroid biosynthetic pathways in *Arabidopsis thaliana* starting from campesterol. Bold red arrows represent the proposed main BR biosynthetic pathway. Black and blue arrows represent the CN-dependent routes. The light blue lines show the late C-6 oxidation pathway and the dark blue lines show the early C6 oxidation pathway

**Fig. 2:**
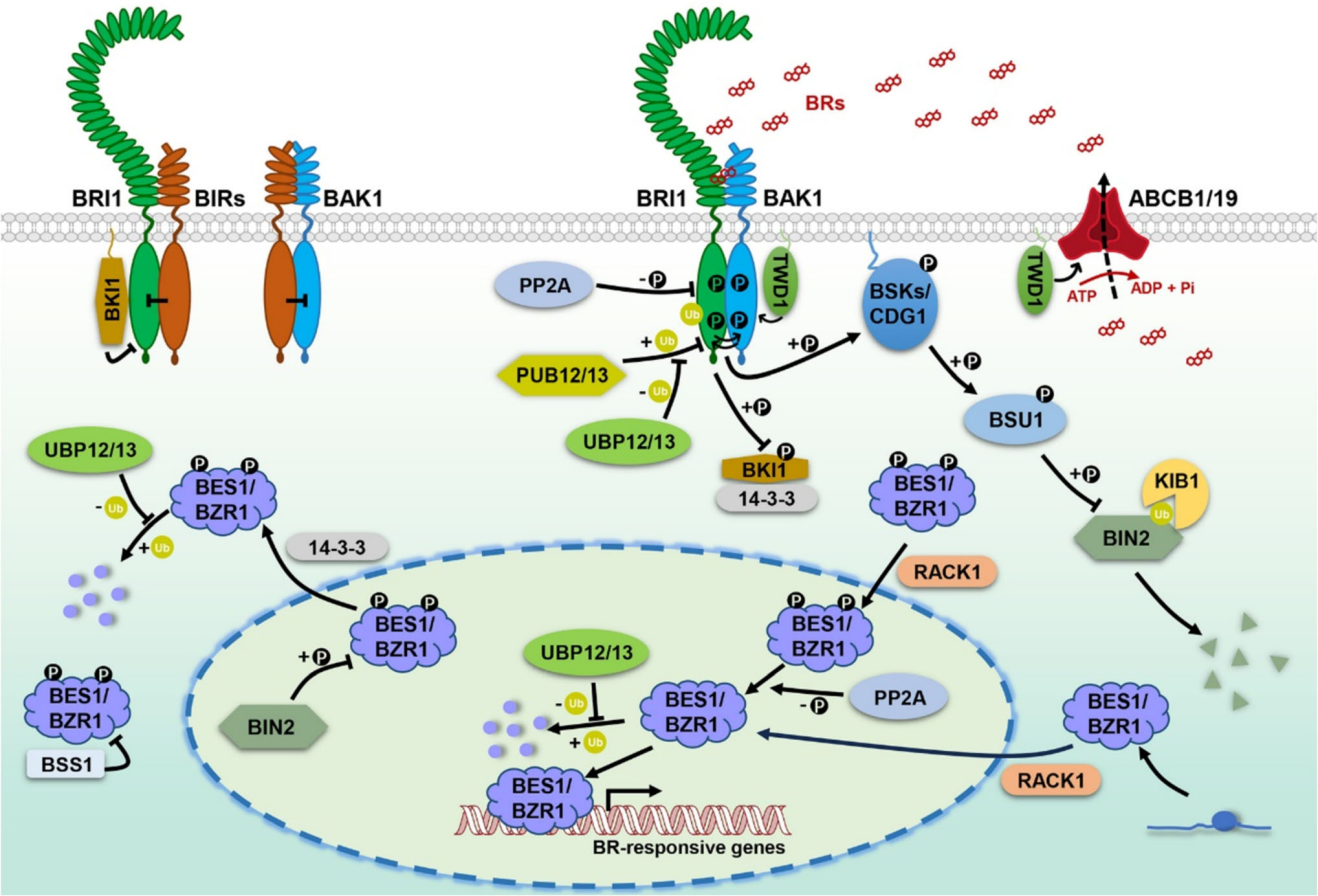
A current model of the BR signaling pathway in *Arabidopsis thaliana*. In the absence of BRs, BRI1 interacts with BKI1 and BIRs, and BAK1 associates with BIRs, inhibiting the formation of the BRI1-BAK1 complex. 14–3-3 proteins promote the export of phosphorylated BES1/BZR1 from the nucleus to cytoplasm. BSS1 helps to retain BES1/BZR1 in the cytosol. When BRs are exported from the cytoplasm to the extracellular matrix via ABCB1/19, they are perceived by the BRI1-BAK1 receptor–coreceptor complex, promoting the dissociation of BKI1 from BRI1. BRI1 phosphorylates and activates receptor-like cytoplasmic kinases, such as BSKs and CDGs. The activated BSKs/CDGs phosphorylate and activate BSU1, which in turn dephosphorylates and inactivates BIN2. Phosphorylated and non-phosphorylated BES1/BZR1 can be transported to the nucleus with the help of RACK1. In the nucleus, the phosphorylated BES1/BZR1 can be dephosphorylated and activated by PP2A. Finally, non-phosphorylated BES1/BZR1 initiates the transcriptional regulation of downstream BR-responsive genes. TWD1, PP2A, PUB12/13, and UBP12/13 can fine-tune the function of the receptor-coreceptor complex. KIB1 can promote the degradation of BIN2, and UBP12/13 can inhibit the degradation of the BES1/BZR1 protein family

Among the BRs, brassinolide (BL) is the final product of the BR biosynthetic pathway in most plant species and is the most active form. Biosynthesis of BL involves complex and grid routes (Fig. 1; Fujioka and Yokota 2003; Zhao and Li 2012; Wei and Li 2020). A specific BR biosynthetic precursor, campesterol (CR), undergoes at least 8 steps to eventually produce BL. CR can be converted to campestanol (CN), which can be transformed into castasterone (CS) through two parallel pathways designated as an early C6 oxidation pathway and a late C6 oxidation pathway (Fujioka and Yokota 2003). The combination of these two pathways was also named a CN-dependent pathway. In addition, CR can also undergo an early C22 oxidation and then converge into the late C6 oxidation pathway through a CN-independent pathway to form CS (Fujioka et al. 2002; Fujita et al. 2006; Ohnishi et al. 2006). Finally, in most species, CS can be further converted to BL via a Baeyer–Villiger oxidation step (Yokota et al. 1990; Suzuki et al. 1993; Kim et al. 2005). Experimental analyses performed on different routes of BL biosynthesis in several species suggest that the CN-independent pathway converged into the late C6 oxidation pathway is likely the predominant route in plants (Fig. 1; Ohnishi et al. 2012, 2018). Recently, it was shown that BRs need to be exported from the cytoplasm to the extracellular region, via plasma membrane-localized transporters, named ABCB1 and ABCB19, before they can be perceived by their receptor and co-receptor (Fig. 2; Ying et al. 2024; Wei et al. 2024). Since 1996, most of the enzymes that catalyze the reactions in the BR biosynthesis pathway have been identified in the model plant *Arabidopsis*, including DWARF4 (DWF4), CONSTITUTIVE PHOTOMORPHOGENESIS AND DWARFISM (CPD), De-ETIOLATED 2 (DET2), ROTUNDIFOLIA 3 (ROT3), and CYP85A1/2 (also known as BR6ox1/2) (Wei and Li 2020) except for the C-2 hydroxylases and C-3 oxidoreductases that are yet to be determined (Fig. 1). Among them, *DWF4* is weakly expressed in fast-growing tissues and DWF4 is considered as a rate-limiting enzyme of BR biosynthesis (Choe et al. 2001; Bancosİ et al. 2002; Kim et al. 2006; Guo et al. 2010). Therefore, *DWF4* is often considered one of the targets for manipulating endogenous levels of BRs (Guo et al. 2010; Chung et al. 2011; Zheng et al. 2018b; Li et al. 2018).

Biosynthesized BL depends on a complete signaling pathway for its biological function. To date, the BR signaling pathway has been extensively studied in *Arabidopsis* (Fig. 2). It was found that BRs are perceived by the extracellular domains of their receptor BRASSINOSTEROID INSENSITIVE 1 (BRI1) and co-receptor BRI1-ASSOCIATED KINASE 1 (BAK1) on the plasma membrane (Li and Chory 1997; Li et al. 2002; Nam and Li 2002; She et al. 2011; Sun et al. 2013). BRI1 and BAK1 belong to the subfamily of cell-surface localized leucine-rich repeat receptor-like kinases (LRR-RLKs), which contain a number of functionally redundant paralogs (Zhou et al. 2004; Caño-Delgado et al. 2004; Gou et al. 2012). The perception of BRs by the receptor and co-receptor complex promotes the interaction and transphosphorylation of the intracellular kinase domains of these two LRR-RLKs via a “double-lock” mechanism (Wang et al. 2008; Jaillais et al. 2011). This process leads to the dissociation of a BRI1 negative regulator, named BRI1 KINASE INHIBITOR 1 (BKI1), from the C-terminus of BRI1, resulting in the full activation of BRI1 (Wang and Chory 2006). Activated BRI1 then phosphorylates and activates downstream membrane-anchored RECEPTOR-LIKE CYTOPLASMIC PROTEIN KINASEs (RLCKs), such as BR SIGNALING KINASE 1 (BSK1) and CONSTITUTIVE DIFFERENTIAL GROWTH 1 (CDG1) (Tang et al. 2008; Kim et al. 2011). Activated RLCKs further phosphorylate a phosphatase BRI1 SUPPRESSOR 1 (BSU1), which in turn dephosphorylates and inactivates a key negative regulator of the BR signaling, BRASSINOSTEROID INSENSITIVE 2 (BIN2), a GSK3-like protein kinase (Kim et al. 2009). Inactivation of BIN2, along with the function of a group of PP2A phosphatases, leads to the accumulation of non-phosphorylated forms (activated) of a transcription factor family, including BRI1-EMS-SUPPRESSOR 1 (BES1) and BRASSINAZOLE RESISTANT 1 (BZR1), in the nucleus, initiating the transcriptional regulation of BR-responsive genes (Wang et al. 2002, 2021a; Yin et al. 2002; He et al. 2005; Tang et al. 2011).

In addition, several other regulators were also found to be involved in the regulation of BR signaling pathway (Fig. 2). For example, in the absence of BRs, BAK1 INTERACTING KINASEs (BIRs) were found to associate with BAK1 and then block the formation of the BRI1-BAK1 complex (Imkampe et al. 2017; Hohmann et al. 2018; Großeholz et al. 2020). 14–3-3 proteins help the accumulation of phosphorylated forms of BES1/BZR1 in the cytoplasm (Gampala et al. 2007). BRZ-SENSITIVE-SHORT HYPOCOTYL 1 (BSS1) inhibits the transport of BES1/BZR1 from the cytosol to the nucleus (Shimada et al. 2015). In the presence of BRs, on the other hand, TWISTED DWARF 1 (TWD1) was found to enhance the interaction and transphosphorylation of BRI1 and BAK1 (Chaiwanon et al. 2016; Zhao et al. 2016). Prohibitin 3 (PHB3) was found to act as a direct substrate of BRI1 and BAK1 to regulate BR signaling (Li et al. 2024). E3 ubiquitin ligase proteins PLANT U-BOX PROTEIN 12/13 (PUB12/13) mediate the degradation of phosphorylated BRI1 (Zhou et al. 2018). UBIQUITIN-SPECIFIC PROTEASE 12/13 (UBP12/13) regulate the de-ubiquitination of both BRI1 and BES1/BZR1 (Luo et al. 2022; Park et al. 2022; Xiong et al. 2022). KINK SUPPRESSED in *bzr1-1D* 1 (KIB1) mediates the degradation of BIN2 (Zhu et al. 2017). RECEPTOR for ACTIVATED C KINASE 1 (RACK1) promotes the nuclear import of BES1/BZR1 protein (Li et al. 2023). And TETRATRICOPEPTIDE THIOREDOXIN-LIKE (TTL) acts as a scaffold protein to optimize BR signaling transduction (Nam and Li 2004; Amorim-Silva et al. 2019). Recent studies indicated that BR signaling via BRI1 directly regulates the activities of H^+^-ATPases in a non-genomic mechanism to rapidly regulate cell elongation (Li et al. 2022b).

## Effects of BR signaling on regulation of fruit development

Fruits are important organs of some horticultural plants, which are developed from the ovaries or other floral parts after fertilization in the angiosperms. In plants, fruits serve multiple functions, such as nourishment, protection, and seed dispersal. In addition, many fruits are requisite to our diet, providing us with essential nutrients, vitamins, and mineral elements. Based on morphology, fruits can be divided into dry fruits and fleshy fruits. Cereal crops and cruciferous fruits belong to the dry fruits, while objects of fruticulture mainly focus on fleshy fruits. In this section, we systematically discuss the regulation of fleshy fruit development by BR signaling.

**Fig. 3:**
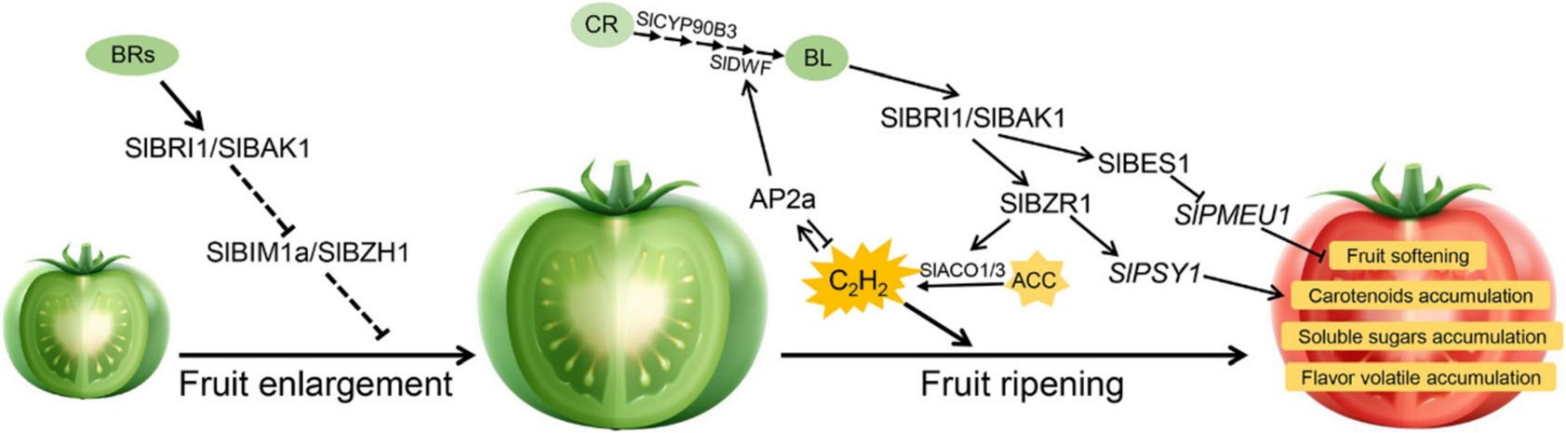
An overview of the BR signaling in regulating tomato fruit development and ripening. BR can promote fruit enlargement by inhibiting the function of the SlBIM1a-SlBZH1 module. During fruit ripening, BR signaling can inhibit the expression of *SlPMEU1* via SlBES1, and promote the expression of *SlPSY1* and *SlACO1/3* via SlBZR1, enhancing various biochemical transformations of organic compound, and ethylene production. In the meantime, ethylene can promote BR biosynthesis, further regulating fruit ripening

Fruit enlargement is a complex process, where BRs were shown to play a significant role. For example, in strawberries (*Fragaria* × *ananassa*), the BR content increases rapidly at the small-green fruit stage (Chai et al. 2013). Considering this influence of BRs, exogenous application of BRs was done in cucumber (*Cucumis sativus*), which exhibited induction of expression of cell cycle-related genes at an early fruit development stage (Fu et al. 2008). Likewise, analyses of the expression patterns of BR biosynthesis genes and BR levels also confirmed the importance of BRs at an early developmental stage of tomato (*Solanum lycopersicum*) fruits (Montoya et al. 2005). Furthermore, targeted modifications of the phosphorylation sites of a receptor of BRs in tomatoes (SlBRI1) resulted in the regulation of fruit development and yield (Wang et al. 2019a, 2021b). Downstream transcription factors of the BR signaling, however, sometimes have different effects on the development of fruits. For instance, in loquat (*Eriobotrya japonica*), EjBZR1 acts as a negative regulator in fruit enlargement because it functions to directly repress the expression of *EjCYP90A*, an enzyme involved in BR biosynthesis (Su et al. 2021). Similarly, SlBZH1 (a homolog of AtBZR1), can interact with BES1-INTERACTING MYC-LIKE1a (SlBIM1a) and negatively regulate fruit growth (Fig. 3; Mori et al. 2021). On the other hand, overexpression of *SlCESTA*, a tomato bHLH-type transcription factor, was identified as a positive regulator of BR biosynthesis, which promoted fruit growth (Shuai et al. 2022). These observations indicate the presence of a sophisticated feedback regulation of BR signaling on fruit growth and development.

Fruit ripening is a developmental process characterized by significant changes in color, texture, aroma, and flavor, which often result from various biochemical transformations of organic compounds. Expression of BR biosynthetic genes and endogenous BR levels were found to increase during grape (*Vitis vinifera*) berry ripening (Symons et al. 2006). In another study, the exogenous application of BRs promoted the biosynthesis of terpenoid aroma components and the accumulation of anthocyanins in grape berries, enhancing the aroma and color of berries (Zheng et al. 2020). Similarly, both ectopic expression of *AtBZR1-1D* and exogenous application of BRs in tomatoes exhibited enhanced accumulation of carotenoid and carbohydrate contents during fruit ripening (Vidya Vardhini and Rao 2002; Liu et al. 2014). Overexpression of *SlDWF* (a tomato gene encoding a BR C6 oxidase) was also shown to accelerate fruit ripening (Li et al. 2016a). Further studies demonstrated that SlBZR1 can directly activate the expression of key carotenoid biosynthesis genes, such as *SlPSY1*, as well as several other genes encoding ripening-related transcription factors, thereby enhancing fruit lycopene content and promoting fruit ripening (Fig. 3; Sang et al. 2022; Meng et al. 2023). In Chinese kale (*Brassica oleracea var. alboglabra*), BR treatment was found promising to promote the accumulation of carotenoids (Zhang et al. 2024a). Further analyses indicated that BoaBZR1.1 can directly activate the expression of *BoaCRTISO* and *BoaPSY2*, two genes associated with the carotenoid biosynthetic pathway (Zhang et al. 2024a). Exogenous application of BRZ (a specific inhibitor of the BR biosynthesis pathway) and down-regulation of *FaBRI1* resulted in delayed fruit red coloration in strawberries (Chai et al. 2013). In contrast to these positive examples, BR signaling was reported to act as a suppressor of the fruit-ripening process in some horticultural plants. This negative effect was because BR signaling can inhibit the accumulation of flavonoids, anthocyanins, and proanthocyanins of apples (*Malus domestica*), via MdBEH2.2 (an ortholog of BES1/BZR1)-MdMYB60 (a MYB transcription factor), MdBZR1-MdJa2 (a MADS-box transcription factor), and MdBZR1-MdCOL6 (a positive regulator of the anthocyanin biosynthesis) regulatory modules (Wang et al. 2021d, 2023b; Su et al. 2022). Under phosphate starvation conditions, MdBIN2 can phosphorylate PHOSPHATE STARVATION RESPONSE1 (MdPHR1, a core regulator of phosphate starvation response), and promote phosphate deficiency‐induced anthocyanin biosynthesis, altering fruit coloring (An et al. 2023a).

Fruit softening is another important ripening process and is primarily attributed to changes in cell wall structure. The cell wall is composed of various components, such as cellulose, hemicellulose, pectin, and structural proteins. In tomatoes, SlBES1 can directly repress the expression of *SlPMEU1*, a gene encoding a pectin methylesterase, inhibiting pectin de-methylesterification and promoting fruit softening (Fig. 3; Liu et al. 2021a). Similarly, the protein level of SlPMEU1 is decreased in transgenic tomatoes overexpressing *AtBZR1-1D*, a constitutively active form of BZR1 (Liu et al. 2016). In contrast, MaBZR1/2 was shown to repress the transcription of several cell wall-modifying genes, via their interaction with MaMPK14, thereby negatively regulating banana (*Musa acuminata*) fruit softening and ripening (Guo et al. 2019; Shan et al. 2020). Likewise, PbBZR1 was also shown to negatively regulate lignin biosynthesis in Chinese white pears (*Pyrus bretschneideri*) (Cao et al. 2020).

During fleshy fruit ripening, BRs can synergistically interact with ethylene, another essential hormone involved in the fruit-ripening process. For instance, either exogenous application of BRs or increasing endogenous BR levels can enhance ethylene production in tomatoes, thereby accelerating fruit ripening (Hu et al. 2020). It was found that SlBZR1 can directly activate *SlACO1/3*, resulting in increased production of ethylene (Fig. 3; Meng et al. 2023). In addition, the ethylene-regulated transcription factor APETALA2a (SlAP2a) can upregulate the expression of *SlDWF* and enhance BR biosynthesis, thus promoting fruit ripening (Fig. 3; Sang et al. 2022). In bananas, exogenous application of BRs was reported to promote fruit ripening by accelerating the expression of ethylene biosynthesis genes, while MaBZR1/2 was recognized as a negative regulator that represses the transcription of ethylene biosynthetic genes (Guo et al. 2019). BR signaling, however, was shown to reduce ethylene production and delay fruit ripening by repressing ethylene biosynthesis in fruits such as litchis (*Litchi chinensis* Sonn.) (Ma et al. 2021), ussurian pears (*Pyrus ussuriensis*) (Ji et al. 2021), and jujubes (*Ziziphus jujuba* cv. Huping) (Zhu et al. 2010). The BES1/BZR1 transcription factor family also exhibits functional differentiation in regulating ethylene production. In persimmons (*Diospyros kaki*), it was observed that DkBZR1 suppresses the transcription of *DkACS1*, while DkBZR2 activates the transcription of *DkACO2*, leading to the inhibition and promotion of ethylene production, respectively (He et al. 2021). These findings highlight the feedback regulatory role of the BES1/BZR1 family in horticultural plants.

## Effects of BR signaling on leaf morphology and biomass production

Leaf morphology, including leaf shape and size, is of great importance in leafy vegetables because it is directly related to consumer choices and economic value. BRs are able to regulate leaf growth by accelerating cell proliferation, cell expansion, as well as cell wall remodeling (Zhiponova et al. 2013; Xiong et al. 2021). An earlier study reported that both BR-deficient and BR-insensitive mutants can alter the leaf phenotypes of *Arabidopsis*, including rounded, crinkled, thickened, twisted, and dark-green rosette leaves (Clouse et al. 1996). In Chinese cabbages (*Brassica rapa* ssp. *pekinensis*), a plasma membrane-associated protein, called OCTOPUS (BrOPS), was identified as a regulator of leaf curvature through a BR-dependent mechanism (Zhang et al. 2022b). Mutations in *BrOPS* displayed an enhanced inward curling phenotype of the leafy heads compared to wild type. Further analysis indicated that BrOPS recruits BrBIN2 to the plasma membrane, promoting the accumulation of non-phosphorylated BrBES1 during an early heading stage (Zhang et al. 2022b). This non-phosphorylated BrBES1 subsequently represses the expression of a leaf polarity-related transcription factor *BrAS1*, regulating the shape of leaves. The regulatory mechanism involving BrOPS and BrBIN2 resembles to that found in *Arabidopsis* (Truernit et al. 2012; Zhang et al. 2022b). The *Brops*-like leaf phenotype, however, was not observed in *Arabidopsis*, suggesting a unique BR-determined morphology in different plant species.

Leaves serve as primary organs for photosynthesis and provide essential materials and energy for growth and crop yields. Numerous studies indicated that BR signaling regulates photosynthesis at various stages (Siddiqui et al. 2018). Exogenous application of BRs was reported to increase chlorophyll content and photosynthesis capacity of cucumbers (Yu 2004; Xia et al. 2009a), cowpeas (*Vigna unguiculata*) (Lima and Lobato 2017), mustards (*Brassica juncea*) (Wani et al. 2019), and peppers (*Capsicum annuum*) (Hu et al. 2013). In tomatoes, BRs can enhance Calvin cycle activity and improve the net photosynthetic rate in a BZR1-dependent manner (Li et al. 2016b; Yin et al. 2022). Chloroplasts, the main compartment for photosynthesis, are also regulated by BRs. In *Arabidopsis*, BR signaling was reported to regulate chloroplast development by repressing the transcription activities of *GOLDEN2-LIKE 1/2* (*GLK1/2*) via BES1, and the protein stability of GLK1/2 through BIN2 (Yu et al. 2011; Zhang et al. 2021). Furthermore, exogenous application of BRs was able to induce stomatal movement in tomato plants (Xia et al. 2014), which in turn affects CO_2_ absorption in leaves and enhances photosynthetic rates.

## Effects of BR signaling on regulating stem development

In plants, stems are responsible for supporting and transporting water, various nutrients, and signaling molecules, with BRs having a significant influence. For example, BR-deficient and BR-insensitive mutants was reported to have a dwarfed stature with compact leaves (Clouse et al. 1996). BR gain-of-function mutants, on the other hand, showed an elongated organ phenotype (Yin et al. 2002). Some BR signaling gain-of-mutants, such as *bzr1-1D*, also displayed organ–fusion phenotypes (Gendron et al. 2012). These observations indicate that BR signaling can significantly alter plant architecture. Mutations in various BR-related genes of several horticultural plants, were found to exhibit a dwarfed phenotype, which include *CsDWF1/CsDWF7/CsDET2/CsSCP-1* (four genes in cucumber encoding a BR C-24 reductase, C5 desaturase, C5 reductase, and C6 oxidase, respectively), *PsLKA* (a homologous gene of *BRI1* in peas, *Pisum sativum*), *PsLK/PsLKB*/*PsLKE* (three genes in pea encoding BR C5 reductase, C24 reductase and C6 oxidase, respectively), and *SlDPY* (a tomato gene encoding a BR C23 hydroxylase) (Koka et al. 2000; Schultz et al. 2001; Nomura et al. 2003, 2004; Jager et al. 2007; Wang et al. 2017; Hou et al. 2017; Zhang et al. 2022a, 2024b). In contrast, transgenic plants overexpressing *SlDWF* showed a slender organ phenotype (Li et al. 2016a). In western pears (*Pyrus communis*), a dwarf variety was obtained due to increased transcription of *ARABINOGALACTAN PROTEIN 7–1* (*PcAGP7-1*, a gene encoding a glycosylated hydroxyproline-rich glycoprotein), which also resulted in reduced levels of BRs (Zheng et al. 2022). It has been reported that PcBES1 and PcBZR1 can directly repress the transcription of *PcAGP7-1* and form a BR-feedback regulatory module, named BL-PcBES1/BZR1-PcAGP-BL, thereby regulating stem growth and plant architecture (Zheng et al. 2022). Likewise, an apple transcription factor MdWRKY9 was found to contribute to the dwarf phenotype of M26 rootstock by directly repressing the transcription of *MdDWF4* and decreasing BR biosynthesis (Zheng et al. 2018b). In moso bamboo (*Phyllostachys edulis*), BR signaling can accelerate stem elongation via repressing the expression of *POACEAE SPECIFIC AND BR RESPONSIVE GENE 1* (*PePSBR1*), a gene encoding a negative regulator in plant growth and development (Guo et al. 2020).

Lateral branches are another important part of plant architecture. BRANCHED1 (BRC1) is a TCP transcription factor, which negatively regulates the outgrowth of buds (Janssen et al. 2014; Wang et al. 2018). SlBZR1 was shown to bind to the promoter of *SlBRC1* and suppress its expression, promoting shoot branching of tomatoes (Xia et al. 2021). In peach (*Prunus persica*), low BR content was reported to cause a reduction in the number of sylleptic branches (Wang et al. 2024b). Further analysis showed that PpTCP14 can transcriptionally suppress the expression of *PpDWARF2*, which is a gene encoding a BR biosynthesis enzyme (Wang et al. 2024b). In addition, miR6288b-3p can also target and cleave *PpTCP14* mRNA, while lncRNA1 acts as an endogenous target mimic of miR6288b-3p and inhibits its cleavage activity (Wang et al. 2024b).

Vascular tissues in stems serve as mechanical supports and transport water and various nutrients. In *Arabidopsis*, *BRI1* was shown to be ubiquitously expressed in various tissues (Caño-Delgado et al. 2004), whereas its two homologous genes, namely *BRI1-LIKE 1* (*BRL1*) and *BRL3*, are specifically expressed in the vascular tissues. Importantly, these three kinases (BRI1, BRL1, and BRL3) were proven to be the receptors of BRs (Caño-Delgado et al. 2004; Zhou et al. 2004). As a result, a *bri1 brl1 brl3* triple mutant exhibited reduced vascular differentiation and unusual formation of phloem fibers in the stem (Caño-Delgado et al. 2004a; Lozano-Elena and Caño-Delgado 2019). Likewise, the roles of BRs in regulating vascular development were confirmed in multiple horticultural plants. For example, the application of high concentrations of BRZ inhibits the development of secondary xylem in cresses (*Phyllostachys edulis*) (Nagata et al. 2001). Similarly, exogenous application of BL was reported to promote the growth of xylem of liriodendrons (*Liriodendron tulipifera*) (Jin et al. 2014). Overexpression of *PtBRI1.2* and *PtCYP85A3* (a poplar gene encoding a BR C6 oxidase) in poplar (*Populus trichocarpa*) resulted in increased xylem thickness, and facilitated shoot growth and wood formation (Jin et al. 2017; Jiang et al. 2021). Another recent study reported that simultaneous mutations of *PdBRI1.1*, *PdBRI1.2*, *PdBRI1.3*, and *PdBRI1.6* caused extremely abnormal stem development of *Populus deltoides* × *Populus euramericana* (Wang et al. 2022). Further analysis indicated that BRs can up-regulate the expression of genes related to secondary cell wall formation through a regulatory module, PtBZR1-PtWNDs (Jiang et al. 2021). Integrative omics analysis of *Pdbri1s* suggests that BRs can also be involved in procambial cell division and xylem differentiation (Wang et al. 2022). Section analysis of multiple BR-deficient and BR-insensitive mutants reconfirmed that BR signaling is necessary for the differentiation of xylem in tomatoes (Lee et al. 2019). It was shown that SlBZR1/2 can activate the expression of *WALLS ARE THIN1* (*SlWAT1*), a tomato tonoplast membrane-localized auxin efflux carrier, thereby enhancing auxin signaling in the vascular cambium and facilitating xylem formation (Lee et al. 2021).

For some horticultural plants, stems undergo metamorphosis to develop into nutrient storage organs, such as the tuber of potatoes (*Solanum tuberosum*). Expression pattern analysis showed that *StBRI1* was highly expressed in potato tubers (Huang et al. 2021; Deng et al. 2024). Similarly, overexpression of *StBRI1* promotes tuber development (Deng et al. 2024). On the other hand, down-regulation of the transcription of *StBRI1* was found to reduce tuberization (Huang et al. 2021). Further analysis revealed that StBRI1 could phosphorylate the penultimate Thr in PHA2 to release its autoinhibition, thereby promoting potato tuber development in a non-genomic manner (Deng et al. 2024).

## Effects of BR signaling on environmental adaptation of horticultural plants

Unlike animals, plants cannot escape environmental stress and must adapt to adverse conditions in order to survive. Consequently, plants have developed various strategies to adapt to constantly changing environments and achieve optimal growth. While BRs were originally identified as a group of growth-promoting phytohormones, recent studies have provided strong evidence that they also play a role in coordinating plant growth and defense (Yu et al. 2018; Nolan et al. 2020; Yao et al. 2023). It was reported that exogenous application of BRs can confer resistance against a diverse spectrum of environmental stresses, including extreme temperatures, drought, salinity, heavy metals, polycyclic aromatic hydrocarbons, pesticide, and pathogenic microbes (Yu et al. 2002; Kagale et al. 2006; Ahammed et al. 2012; Yin et al. 2016; Sharma et al. 2022). A few recent studies have suggested that BRs can enhance the adaptability of horticultural plants to stress conditions. For instance, the treatment of cucumbers with BRs was shown to elevate reactive oxygen species (ROS) levels, which resulted in prompting systemic stress tolerance (Xia et al. 2009b). Similarly, BRs were shown to induce the accumulation of ROS in tomatoes, which subsequently activated the MAPK cascade and enhanced resistance to root-knot nematodes (Song et al. 2018).

The BES1/BZR1 family members are crucial for regulating downstream target genes and maintaining a balance between plant growth and adaptation. In tomatoes, SlBZR1 was reported to directly activate the transcription of *RESPIRATORY BURST OXIDASE HOMOLOG1* (*SlRBOH1*), a gene encoding a key component of ROS signaling (Fang et al. 2019). In Chinese cabbages, all 15 members of the BrBES1/BrBZR1 family display differential expression patterns in response to various abiotic stresses (Saha et al. 2015). Similarly, the expression of *VvBES1* in grapes was found to respond to certain abiotic stresses (Cao et al. 2023a). Transgenic *Arabidopsis* plants overexpressing *VvBES1-3* showed elevated salt stress tolerance. In apples, MdBES1 showed multifaceted functions under different stress conditions (Liu et al. 2021b). MdBES1 acts as a positive regulator during cold stress and pathogen attack, while it serves as a negative regulator under drought conditions. This complex regulatory module is mainly achieved through the direct promotion or inhibition of the expression of *MdMYB88* (a core regulator in plant stress adaptation responses) by MdBES1 under cold stress and pathogen attack, or drought conditions, respectively (Liu et al. 2021b). In addition, MdBZR1 can regulate the ABA response by directly inhibiting the expression of *ABSCISIC ACID-INSENSITIVE5* (*MdABI5*), a downstream transcription factor of ABA signaling (Liu et al. 2021c). Moreover, MdBZR1 and MdBES1 can directly induce the expression of *MdGA20OX2* and *MdGA3OX1* (two genes encoding gibberellin biosynthesis enzymes) in a BR-dependent manner, thereby enhancing salt tolerance of apple tree (Wang et al. 2019b). Under nitrogen starvation, BR signaling has been shown to promote autophagy, via upregulating the expression of *autophagy-related genes* (*ATGs*) via SlBZR1, accelerating cellular nutrient recycling in tomatoes (Wang et al. 2019c).

Temperature is one of the main factors affecting the growth and development of plants. Under an optimal temperature condition, BRs were reported to induce the expression of *CRABS CLAW a* (*SlCRCa*, a vital regulator of floral meristem termination in tomatoes), which can directly inhibit the expression of *WUSCHEL* (*SlWUS*), thereby terminating floral meristem activity and ensuring normal shape of tomato fruits (Wu et al. 2024). Under heat stress conditions, the transcriptional repressor EARLY FLOWERING3s (SlELF3s) was reported to inactivate the expression of BR biosynthetic genes to interfere with BR hemostasis in the floral meristem, resulting in malformed tomato fruits (Wu et al. 2024). On the other hand, high temperature but below the stress conditions can induce thermos-morphogenesis of plants. A bHLH-type transcription factor, PHYTOCHROME-INTERACTING FACTOR4 (PIF4), was shown to play a critical role during this process (Quint et al. 2016). It was also shown that SlPIF4 can directly up-regulate the expression of *SlDWF*, promoting BR biosynthesis and hypocotyl elongation of tomato seedlings (Zhu et al. 2024). Under low-temperature conditions, SlBZR1 can activate the expression of *C-REPEAT BINDING FACTORs* (*SlCBFs*), and *9‐CIS‐EPOXYCAROTENOID DIOXYGENASE1* (*SlNCED1*, a gene encoding a key ABA biosynthesis enzyme), thus enhancing tolerance of tomato to low-temperature (Fang et al. 2021; An et al. 2023b). In peaches, PpBZR1 showed an enhanced tolerance of fruits to cold stress via suppressing sucrose degradation (Zhang et al. 2023). For perennial plants, dormancy is a strategy to overcome seasonal environmental changes and ensure normal growth and development at the right time. For example, gibberellins, BRs, and jasmonic acid was reported to promote bud growth in Asian pear (*Pyrus pyrifolia*) after dormancy (Wang et al. 2024a). Further analysis showed that PpyBZR2 can interact with MYELOCYTOMATOSIS 2 (PpyMYC2) and promote the transcription of *PpyGA20OX1L1*/*PpyGA20OX2L2*, thereby releasing pear bud dormancy (Wang et al. 2024a). In addition, PpyBZR2 can directly repress the expression of *DORMANCY-ASSOCIATED MADS-box 3* (*PpyDAM3*, a Rosaceae specialized *SHORT VEGETATIVE PHASE-LIKE* genes that positively regulate bud dormancy), thereby promoting pear bud breaking (Wang et al. 2024a).

BRs were also shown to play essential roles in the growth and development of roots (Wei and Li 2016). However, most relevant studies were carried out on the model plant *Arabidopsis*, despite some investigations being carried out on horticultural plants. For example, in sugar beet (*Beta vulgaris*), exogenous application of BRs stimulated the development of root parenchyma cells and secondary xylem, increasing root diameter (Wang et al. 2021c). Overexpressing *SlBRI1* showed an increase in root length and lateral root emergence (Nie et al. 2017). A similar role of BRs in stimulating lateral root formation was also observed in apples (Zheng et al. 2018a). Adventitious roots forming on stems or leaves can also be induced by exogenous application of BL in cucumbers, which was probably achieved by up-regulating the production of endogenous nitric oxide (Li et al. 2020). Based on these findings, it can be noted that a well-developed root system can inherently enhance plant productivity and strengthen its resilience against adverse environmental conditions. Therefore, manipulating BR signaling is imperative for molecular breeding in horticultural plants to improve root development and environmental adaptability.

## Conclusion and perspectives

**Fig. 4:**
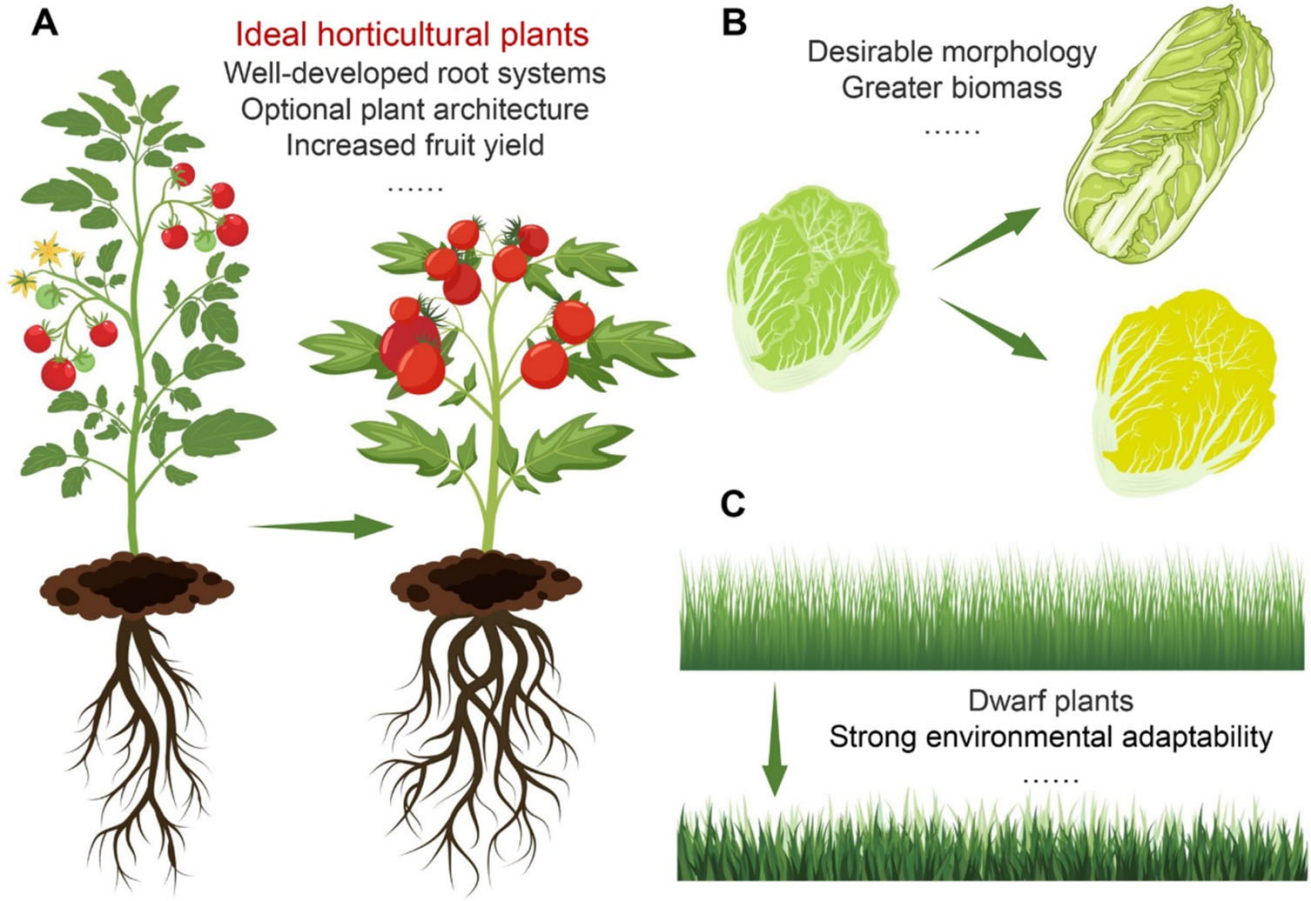
Schematic representation of achievable horticultural plant breeding objectives via altering the BR signaling pathway. **A**. The ideal horticultural crops are those with well-developed root systems, optimal plant architecture, high yield of fruits, etc. **B**. The breeding goal of leafy vegetables is to obtain a plant with desirable leaf morphology and higher biomass. **C**. The ideal turfgrasses are those with dwarfed stature and strong environmental fitness

The objective of plant breeding is to combine multiple desirable traits in a single variety. Studies conducted on model plants and a range of horticultural plants have demonstrated the feasibility of genetically modifying BR signaling to obtain ideal horticultural plants with target properties, such as robust root systems, optimal plant architecture, and high yield (Fig. 4A). In addition, different horticultural plants have various expectations of economic value. For instance, commercially important leafy vegetables are those with diverse leaf morphology and higher biomass (Fig. 4B), whereas ideal turfgrasses are those with dwarfed stature and strong environmental tolerance (Fig. 4C).

Plant hormones, including BRs, are often involved in regulating plant growth and development. Globally altered hormonal signals often result not only in desirable phenotypes for a target organ but also undesirable phenotypes for other organs. A tissue-specific alteration in hormonal signaling can be used to avoid undesirable phenotypes. An earlier study using grafting experiments in peas revealed that BRs cannot be transported over long distances (Symons and Reid 2004). This finding suggests that local modulation of BR homeostasis can be considered feasible to obtain organ-specific traits. Recently, it was reported that tissue-specific reduction of BR signaling in the secondary branch meristems of rice can lead to clustered grains without affecting grain size, which significantly increased crop yield (Zhang et al. 2024c). Such an approach was also used in horticultural plants to achieve desirable traits, such as clustered peppers and roses (Zhang et al. 2024c).

Researchers carried out genetic and structural analyses that revealed some amino acid residues in BRI1, BAK1, or other key components, display unique functions to the BR signaling pathway (She et al. 2011; Sun et al. 2013; Wang et al. 2019a, 2021b). These residues can be the targets for site-direct mutagenesis to either enhance or reduce the BR signaling cascade. In some horticultural plants, members of the BES1/BZR1 family were reported to regulate specific traits by modulating the expression of a set of downstream genes (Table 1), suggesting that targeted alteration in the downstream genes of the BES1/BZR1 subfamily can be another approach to specifically regulate some traits in horticultural plants. In addition, a semi-dwarf wheat cultivar with high nitrogen utilization efficiency was obtained by partially attenuating BR signaling and enhancing gibberellin signaling (Song et al. 2023). This finding suggests that the synergistic regulation of BR and other phytohormone signaling pathways can be used as an alternative strategy for the improvement of horticultural plants via molecular breeding.

**Table 1:** The targeted genes of BES1/BZR1 family homologs in horticultural plants

Common name	Species	BES1/BZR1 family homologs	Targeted genes	Regulatory mechanism	Functions	References
Tomatoes	*Solanum lycopersicum*	SlBES1	*SlPMEU1*	Repression	Inhibiting pectin de-methylesterification and promoting fruit softening and ripening	Liu et al.(2021a)
		SlBZR1	*SlPSY1*	Activation	Enhancing fruit lycopene content	Sang et al.(2022); Meng et al.(2023)
		SlBZR1	*SlACO1/3*	Activation	Increasing ethylene production and promoting fruit ripening	Meng et al.(2023)
		SlBZR1	*SlRBOH1*	Activation	Promoting hydrogen peroxide production and enhancing tolerance to chilling stress	Fang et al.(2019)
		SlBZR1	*SlATG2/6*	Activation	Promoting autophagosome formation and enhancing the tolerance to nitrogen starvation	Wang et al.(2019c)
		SlBZR1	*SlCBFs and SlNCED1*	Activation	Enhancing plant low-temperature tolerance	Fang et al.(2021); An et al.(2023b)
		SlBZR1	*SlBRC1*	Repression	Regulating shoot branching	Xia et al.(2021)
		SlBZR1/2	*SlWAT1*	Activation	Facilitating xylem differentiation and subsequent wood formation	Lee et al.(2021)
Apples	*Malus domestica*	MdBZR1	*MdABI5*	Repression	Inhibiting ABA response	Liu et al.(2021c)
		MdBZR1& MdBES1	*MdGA20OX2* and *MdGA3OX1*	Activation	Promoting gibberellin production and enhancing salt tolerance	Wang et al.(2019b)
		MdBES1	*MdMYB88*	Activation	Enhancing the tolerance to cold stress and pathogen attack	Liu et al.(2021b)
		MdBES1	*MdMYB88*	Repression	Making plant more sensitive to drought stress	Liu et al.(2021b)
		MdBEH2.2	*MdFLS and MdLAR&MdMYB9/11*	Repression	Inhibiting flavonoid biosynthesis and red-flesh coloration	Wang et al.(2021d)
Ussurian pears	*Pyrus ussuriensis*	PuBZR1	*PuACO1 and PuACS1a*	Repression	Reducing ethylene production and delaying fruit ripening	Ji et al.(2021)
Western pears	*Pyrus communis *L.	PcBES1/BZR1	*PcAGP7-1*	Repression	Regulating cell morphogenesis and plant stem growth	Zheng et al.(2022)
Asian Pears	*Pyrus pyrifolia*	PpyBZR2	*PpyDAM3*	Repression	Promoting pear bud breaking	Wang et al.(2024a)
Bananas	*Musa acuminata*	MaBZR1/2	*MaEXP2 and MaPL2 and MaXET5*	Repression	Modifying the cell wall matrix polysaccharide structure and inhibiting fruit softening and ripening	Shan et al.(2020)
		MaBZR1/2	*MaACS1 and MaACO13/14*	Repression	Inhibiting ethylene production	Guo et al.(2019)
Persimmons	*Diospyros kaki*	DkBZR1	*DkACS1*	Repression	Inhibiting ethylene production	He et al.(2021)
		DkBZR2	*DkACO2*	Activation	Promoting ethylene production	He et al.(2021)
Chinese kales	*Brassica oleracea var. alboglabra*	BoaBZR1.1	*BoaCRTISO* and *BoaPSY2*	Activation	Inducing carotenoid biosynthesis	Zhang et al.(2024a)
Chinese cabbages	*Brassica rapa* ssp. *pekinensis*	BrBES1	*BrAS1*	Activation	Regulating leaf shape formation	Zhang et al.(2022b)
Loquats	*Eriobotrya japonica*	EjBZR1	*EjCYP90A*	Repression	Regulating cell size and fruit growth	Su et al.(2021)
Poplars	*Populus trichocarpa*	PtBZR1	*PtWNDs*	Activation	Promoting shoot growth and wood formation	Jiang et al.(2021)

Nowadays, although manipulating BR signaling pathway to genetically improve horticultural crops holds great promise, some challenges in the application of biotechnology are hampering the molecular breeding processes. With the rapid development of genome-editing technology, modern molecular breeding can be used to alter plant genomes through precise deletion or insertion of DNA segments, base transversions, or chromosome rearrangement (Li and Xia 2020; Gao 2021; Li et al. 2022a). The existing genome-editing tools, however, are not fully compatible with different plant species. Hence, developing an efficient gene-editing system is important for horticultural plants, such as using an effective promoter to drive the expression of genome-editing components, a clustered regularly interspaced short palindromic repeats (CRISPR) associated nucleases and a guide RNA (Niu et al. 2023). In addition, most horticultural plants are difficult to regenerate, via tissue culture, which is another roadblock to the comprehensive application of genome-editing technology.

Recently, a number of gene delivery systems have been successfully applied in non-model plants. For instance, a transient delivery system, via engineering RNA virus vectors of tomato spotted wilt virus, can be used to deliver a series of large CRISPR/Cas nuclease to different varieties of horticultural plants (Liu et al. 2023; Shen et al. 2024). By grafting wild-type shoots onto transgenic *Arabidopsis* rootstocks containing genome-editing components fused to tRNA-like sequence motifs (TLS), *Cas9* RNA and *gRNA* can be transported from transgenic roots to aerial fruits and seeds, thereby achieving gene editing in *Brassica rapa* (Yang et al. 2023a). Another efficient strategy, named the Cut-dip-budding (CDB) delivery system, showed promise for transforming explants with *Agrobacterium rhizogenes* and achieving heritable gene-editing in a variety of plants possessing strong regenerative capacity (Cao et al. 2023b, 2024; Lu et al. 2024). In conclusion, the development and application of new technologies should continue to be a research priority for successful molecular breeding in horticultural plants.

## Data Availability

Data sharing not applicable to this article as no datasets were generated or analyzed during the current study.
